# Effects of nutrient injection on the Xinjiang oil field microbial community studied in a long core flooding simulation device

**DOI:** 10.3389/fmicb.2023.1230274

**Published:** 2023-10-12

**Authors:** Wei Cheng, Huiqiang Fan, Yuan Yun, Xueqing Zhao, Zhaoying Su, Xuefeng Tian, Dakun Liu, Ting Ma, Guoqiang Li

**Affiliations:** ^1^Key Laboratory of Molecular Microbiology and Technology, Ministry of Education, College of Life Sciences, Nankai University, Tianjin, China; ^2^Tianjin Engineering Technology Center of Green Manufacturing Biobased Materials, Tianjin, China

**Keywords:** MEOR, microbial community, community structure, nutrition, food chain

## Abstract

Microbial Enhanced Oil Recovery (MEOR) is an option for recovering oil from depleted reservoirs. Numerous field trials of MEOR have confirmed distinct microbial community structure in diverse production wells within the same block. The variance in the reservoir microbial communities, however, remains ambiguously documented. In this study, an 8 m long core microbial flooding simulation device was built on a laboratory scale to study the dynamic changes of the indigenous microbial community structure in the Qizhong Block, Xinjiang oil field. During the MEOR, there was an approximate 34% upswing in oil extraction. Based on the 16S rRNA gene high-throughput sequencing, our results indicated that nutrition was one of the factors affecting the microbial communities in oil reservoirs. After the introduction of nutrients, hydrocarbon oxidizing bacteria became active, followed by the sequential activation of facultative anaerobes and anaerobic fermenting bacteria. This was consistent with the hypothesized succession of a microbial ecological “food chain” in the reservoir, which preliminarily supported the two-step activation theory for reservoir microbes transitioning from aerobic to anaerobic states. Furthermore, metagenomic results indicated that reservoir microorganisms had potential functions of hydrocarbon degradation, gas production and surfactant production. Understanding reservoir microbial communities and improving oil recovery are both aided by this work.

## 1. Introduction

Microbial Enhanced Oil Recovery (MEOR) leverages the proliferative and metabolic capacities of microorganisms to enhance oil production (Liu et al., 2010). This approach integrates biotechnological solutions to address challenges in the oil sector. MEOR mechanisms encompass the biological blockage of sizable pores and the deployment of bio-derived surfactants, emulsifying agents, solvents, and gases to modify crude oil attributes, thereby optimizing oil recovery (Niu et al., 2020). Notably, MEOR offers economic viability, minimal reservoir disruption, and environmental sustainability (Patel et al., 2015). Hence, the scientific community is increasingly delving into the microbial community within oil reservoirs (Sharma et al., 2023).

Oil reservoirs can be analogized as vast bioreactors. Prior explorations had characterized them as intricate ecosystems teeming with diverse microbial entities (Orphan et al., 2000). Contemporary understanding demarcates a two-step activation process for reservoir microbes: an initial aerobic phase followed by a sequential facultative anaerobic to fully anaerobic phase (Yu et al., 2012). Within this MEOR framework, native microbes establish a hierarchical “food chain” in oil reservoirs. This “food chain” theory posits that as oil production progresses, dissolved oxygen permeates into the reservoir with the injected water, fostering an aerobic zone in the injection well. Hydrocarbon-oxidizing bacteria (HOB) and other aerobic decomposers, using petroleum hydrocarbons as substrates, get stimulated. Metabolic by-products of HOB, including organic acids and biosurfactants, are then carried over to intermediary and anaerobic regions by the water influx (Spormann and Widdel, 2000; Fan et al., 2022). These compounds are subsequently metabolized by facultative anaerobic nitrate-reducing bacteria (NRB) and anaerobic fermenting bacteria (FMB), leading to the production of smaller organic acids, hydrogen, and alcohols. These in turn serve as energy substrates for sulfate-reducing bacteria (SRB) and methanogens (Varjani and Gnansounou, 2017). Collectively, these microbes orchestrate intricate biogeochemical pathways deep beneath the Earth’s surface (Head et al., 2003).

A comprehensive understanding of the microbial community across different oil production wells within a reservoir block is crucial to enhancing oil yield. Prior research, such as Tang et al. (2012), employing 16S rRNA gene profiling, scrutinized the microbial landscapes in specific blocks of the Huabei Oilfield. Distinct microbial profiles, correlating with differential oil yields, were observed across individual production sites within the same block. Comparative studies by Skidmore et al. (2005) between injected and produced waters in oil reservoirs found substantial bacterial community divergences. Moreover, Chai et al. (2018) unveiled pronounced microbial community disparities even between interconnected wells within the Luliang Oilfield. Factors such as extensive well spacing and the reservoir’s porous nature might be contributing to these observed variances. While recent inquiries suggested minimal influence of injected water on microbial community structures in production wells, broader environmental variables like temperature and pH seem to exert significant influences (Xu et al., 2023). Despite these preliminary findings, the chief determinants and their relative impacts remain elusive.

In this study, we built an 8 m long core microbial oil flooding simulation device to study the dynamic changes of the microbial community structure in the Qizhong Block, Xinjiang oil field. Utilizing 16S rRNA gene sequencing and metagenomic techniques, we closely monitored microbial community transitions following nutrient injection, fostering a deeper understanding of microbial community dynamics in oil wells.

## 2. Materials and methods

### 2.1. Medium and fluids

A basic nutrient mix was employed to promote the growth of indigenous microbes. The nutrient formulation and concentrations are shown in Table 1. All fluids used in this study, including crude oil (the oil phase of the fluid produced from the well) and produced water (the water phase of the fluid produced from the well), were obtained from the Qizhong Block oil field in Karamay, Xinjiang, China. The produced water used in the experiment was mixed with formation-water from 5 production wells. The sample well numbers were 72,602, 72,604, 72,648, 72,649, 72,659. The sample was completely filled in a 15 L sterilized bucket that was previously swept with nitrogen to prevent oxygen from diffusing into the sample. The sample was then immediately transported to our laboratory. All freshly produced liquids were either immediately employed or refrigerated at 4°C for later experiments. The viscosity of crude oil was 5.55 mpa.s (thin oil, 37°C). Ionic composition of the produced water is detailed in Supplementary Table S1.

**Table 1:** Nutrient formulation and concentration for 50 days.

Injection time	Nutrient concentrations (g/L)
1–30 days	Molasses 3.50 Corn extract powder 1.50 NaNO_3_ 6.0 (NH_4_)_2_HPO_4_ 3.0
30–50 days	Molasses 1.75 Corn extract powder 0.75 NaNO_3_ 3.0 (NH_4_)_2_HPO_4_ 0.6

### 2.2. Long core microbial flooding simulation device

The device incorporated four components: a 2 PB series advection pump, intermediate container, an 8 m long sand-filled tube, and a large temperature incubator. The advection pump, operating on the piston mechanism, channeled nutrient solutions from the intermediate container to the sand-filled tube. Two intermediate containers were present, each dedicated for crude oil and the nutrient solution, respectively. The sand-filled tube, constructed from stainless steel, featured 5 linear sand tubes and 4 U-shaped tubes, each equipped with a sampling site and a valve. The overall size of the sand-filled tube was D5 cm × L800 cm. A scheme of the long core microbial flooding simulation device is shown in Supplementary Figure S1. Quartz sand (30 ~ 50 μm) served as the porous medium within the tube, ensuring no voids. The gas permeability was gaged at 4831 millidarcy (md). The formation-water was fully saturated, and the pore volume (PV) had been computed to be 7,040 cm^3^.

The procedure of the flooding experiments aimed at mimicking the entire oil recovery process in field, including the stages of water saturation, oil saturation, water flooding and microbial flooding (tertiary oil recovery). The sand-filled tube was first saturated with formation-water under vacuum conditions (water saturation stage). Secondly, oil was injected into the sand-filled tube until the end of the uniform flow of crude oil (total saturated oil 4,550 cm^3^). After that, the formation-water was injected for the secondary recovery stage (water drive stage). When the water content of the end of the sand-filled tube exceeded 98%, the injection was stopped. Subsequently, the microbial flooding stage was performed. Nutrient solution was injected for water flooding. All tests maintained a constant flow rate of 1 mL/min at 37°C and persisted for 50 days. The volume of released oil and water cut were measured. Oil recovery efficiency (ORE, %) was calculated as follows (Gao et al., 2020):


(1) $$\mathrm{ORE}\left(\%\right)=\frac{\mathrm{Total}\;\mathrm{volume}\;\mathrm{of}\;\mathrm{oil}\;\mathrm{recovery}\;}{\mathrm{Original}\;\mathrm{oil}\;\mathrm{in}\;\mathrm{place}}\times 100$$

“Original oil in place” (mL) is the volume of water displaced by oil saturation.

Water cut (%) was derived as follows:


(2) $$\mathrm{Water}\;\mathrm{cut}\left(\%\right)=\frac{\mathrm{Volume}\;\mathrm{of}\;\mathrm{water}\;}{\mathrm{Volume}\;\mathrm{of}\;\mathrm{production}\;\mathrm{liquid}}\times 100.$$

### 2.3. Sample collection

Throughout this study, the intermediate container’s medium was refreshed bi-daily. Samples were taken from seven sampling sites. Each time, the switch of sampling site was turned on to drain the stored liquid. Samples were collected in a 5 mL centrifuge tube and centrifuged immediately (12,000 r, 10 min). Precipitates catered to genomic DNA extraction, while supernatants contributed to nutrient concentration determination. DNA extraction employed the AxyPrep ™ Genomic DNA Miniprep Kit (Axygen Biosciences, CA, United States), with procedures elaborated in Supplementary material.

### 2.4. Determination of nutrients

Total sugar was performed through the phenol-sulfuric acid method (Liu et al., 2011), with the standard curve presented in Supplementary Table S2. Total nitrogen was gaged *via* the persulfate oxidation approach (Lima et al., 1997), whereas total phosphorus was assessed by the digestion-molybdenum-antimony method, with the required kits supplied by Tianjin Hasi Water Analysis Instrument Co., LTD. Detailed procedures reside in the Supplementary material.

### 2.5. High-throughput 16S rRNA gene sequencing

Genomic DNA was extracted as previously mentioned (Gao et al., 2018). High-throughput sequencing of 16S rRNA genes was performed by Beijing Novogene Co., Ltd. All 16S rRNA genes were sequenced in QIIME2 (Quantitative Insights into Microbial Ecology) 2) according to the methods recommended in the environment for processing.^1^ The double-ended sequences were spliced, and then quality control was carried out to remove primers and barcodes. In the Silva database, we checked the OTUs species information (Quast et al., 2012). The community structure of each sample was counted at the genus level. The α-diversity of the samples was analyzed using the qiime diversity function of QIIME2.^2^ Principal Component Analysis (PCA) was used to analyze the difference of OTU composition in different sampling sites and sampling times. We used “Canoco 5” software for the redundancy analysis (RDA) of each sample. The network diagram between nutrients and microorganisms (OTU) was constructed. Network visualization and topological properties were conducted using R v3.6.3 (“psych” package) and the Gephi platform v0.9.2 (Yun et al., 2021).

### 2.6. Metagenome sequencing analysis

To explore the function of the indigenous microbial community in the long core microbial flooding simulation device, the samples 2–21, 5–20 and 10–20 were selected for metagenomic sequencing, which was completed by Beijing Novogene Co., Ltd. Following weight removal and quality control, we integrated the metagenomic sequencing data with the species classification information, as well as the integrity and pollution degrees of all MAGS obtained. MAGS with Q50 (integrity value −5 × pollution degree) greater than 50 were identified as qualified MAGS, and the MAGS in each sample were screened for subsequent analysis (Yang et al., 2023).

Prodigal software was used to translate the genomic information into protein sequences, and the functional genes and metabolic pathway information contained in each MAG were obtained by comparison with the KEGG database. The metabolic information of MAG with sufficient quality was mapped online, and the key genes in the pathway of alkane metabolism, nitrogen metabolism, sulfur metabolism and methane generation were analyzed. The key genes of alkane metabolism were selected as alkanes monooxygenase *alkB* gene and long chain monooxygenase *ladA* gene (Yun et al., 2021). In nitrogen metabolism, nitrate reduction pathway and denitrification pathway were the main concerns, and the genes involved were *napA*, *nirK*, *nosZ*, etc. Sulfur metabolism was primarily focused on the sulfate reduction pathway with *dsrA* and *dsrB* being the relevant genes involved (Yao et al., 2023). The key gene in the process of methane generation was methyl-coenzyme M reductase *mcrA* gene (Egger et al., 2016).

## 3. Results and discussion

### 3.1. Microbial flooding simulation and nutrient monitoring

**Figure 1:**
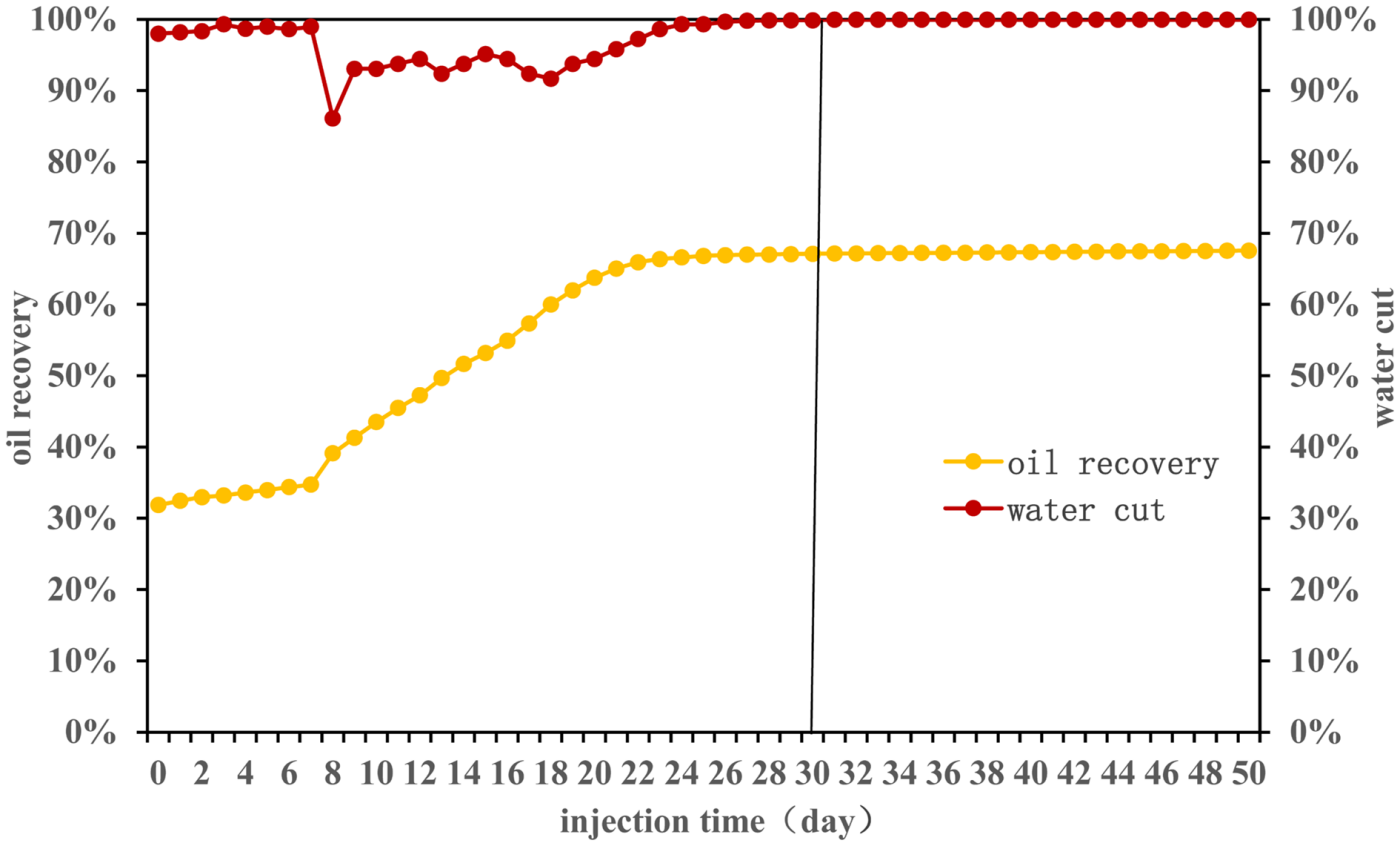
Water cut and oil recovery efficiency during the 50-day monitoring period.

Over a 50-day experimental period, nutrients were injected according to the Table 1. The effects on water cut and oil recovery are delineated in Figure 1. Primary water flooding led to a recovery efficiency nearing 32%. Following nutrient injection, the first 7 days witnessed minimal changes in oil recovery efficiency (ORE). However, between the 7th and 22nd days, ORE surged from 35 to 66%. Post day 23, stability was observed with a final ORE of 68%. It may be because the long-term nutrient injection caused the formation of large pores inside the sand-filled tube. Subsequent nutrients may flow out along the large pores (Chai et al., 2018). Consequently, nutrients could not permeate oil-rich areas, stagnating the ORE.

**Figure 2:**
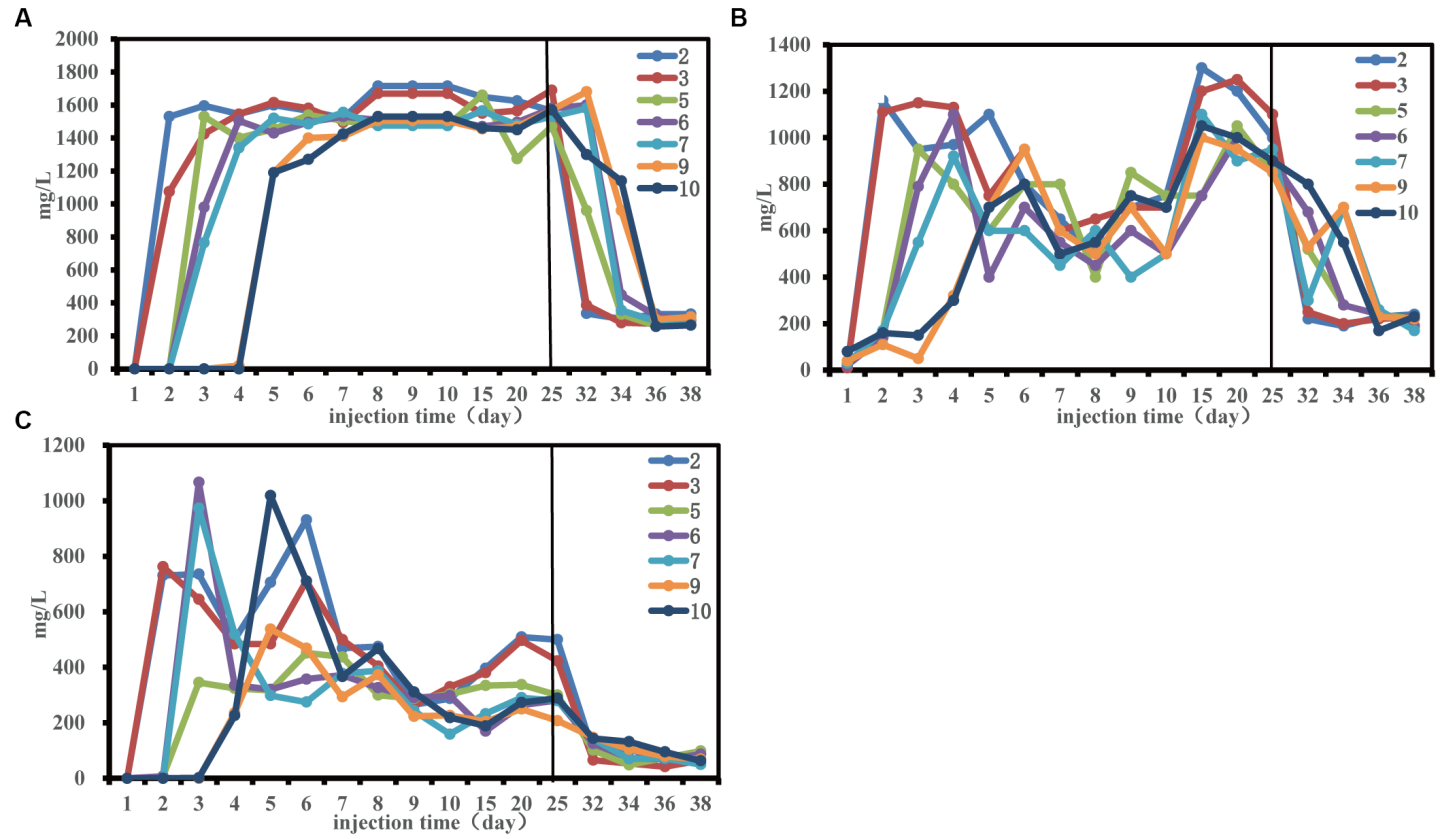
Nutrient concentration changes during the 50-day monitoring period. Concentration of total phosphorus **(A)**, concentration of total nitrogen **(B)** and concentration of total sugar **(C)**.

The change trend of total sugar (TS), total phosphorus (TP) and total nitrogen (TN) at each sampling site was consistent (Figure 2). Notably, there was a delay in the alteration of nutrient concentration at the remote end of the sand-filled tube, requiring a specific period for nutrients to move towards the depths of the tube. This may be the reason that affected the community structure of each sampling site in the early stage.

From the fifth day, residual phosphorus levels stabilized at about 1,500 mg/L (Figure 2A). A noticeable decline in total nitrogen content transpired between the 6th and 15th days, possibly linked to the enhanced ORE (Figure 1). Investigating the interplay between environmental variables and microbial communities, a redundancy analysis (RDA) was conducted, exploring the initial 30 days for effects of sugars, nitrogen, and phosphorus on the community structure at three distinct sampling sites (Supplementary Figure S2 and Supplementary Table S3). Total phosphorus had significant effects on the community structure at the front and middle end of the sand-filled tube. Total nitrogen and total phosphorus had significant effects on the community structure of the terminal sampling site. The results indicated that nutrition had an effect on microbial community structure, which was consistent with findings by Gralka et al. (2020).

### 3.2. PCA and α-diversity analysis of microbial communities

The Shannon index was used to analyze α**-**diversity at each sampling site (Supplementary Figure S3). The α**-**diversity of bacterial community in the two sampling sites was almost the same. From 1 to 30 days, the α**-**diversity in the produced fluid decreased rapidly after nutrient migration to each sampling site. This phenomenon may result from the nutrients injection that significantly stimulated a limited number of bacteria with oil recovery capabilities, causing the decline or vanishing of other microorganisms with diverse functions. Consequently, the species richness of the ecosystem decreases, leading to a reduction of α-diversity (Yun et al., 2021). After reducing the nutrient concentration, α**-**diversity increased slightly, but it was still lower than before the nutrient injection (Supplementary Figure S3), which might be due to the decrease of nitrate concentration in nutrients leading to the increase of α-diversity in bacterial community (Xu et al., 2023).

**Figure 3:**
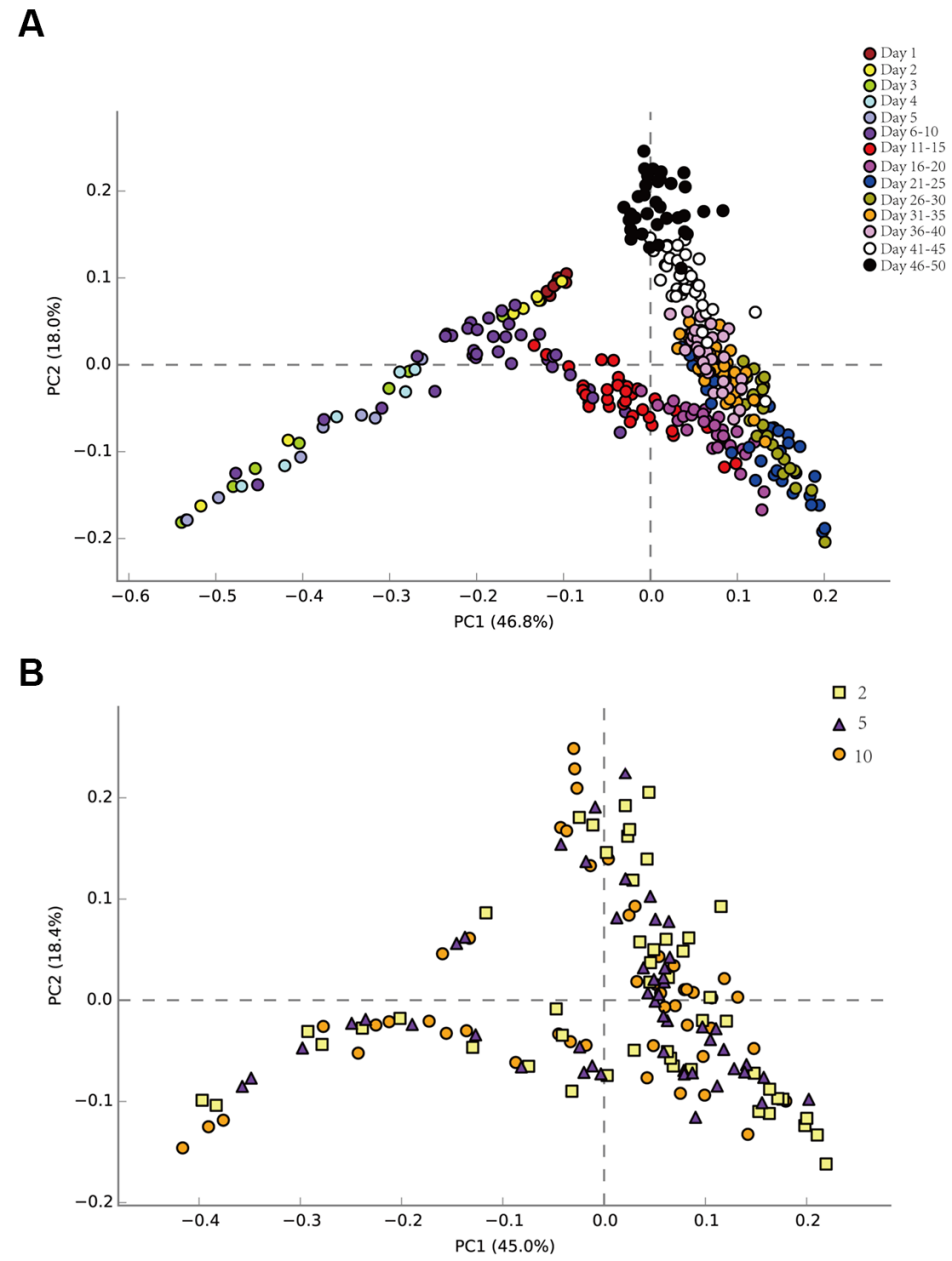
Bacterial principal component analysis (PCA) on the time scale of the produced liquid at each sampling site **(A)**. Bacterial principal component analysis (PCA) on the spatial scale of the produced liquid at sampling sites 2, 5, and 10 **(B)**.

Principal component analysis (PCA) elucidated temporal and spatial bacterial community dynamics (Figure 3). Temporally, the pre-injection phase demonstrated negligible microbial variations (Figure 3A). From 1 to 5 days (about 1 PV), there were some differences in the community structure among the seven sampling sites. The succession direction of the community at the back sampling site was consistent with that at the front sampling site, but later than that at the front sampling site. This may be due to the time it took for nutrients to migrate to the sampling site. Across 6–50 days, community structure stability was evident. The community succession direction was consistent on the time scale. Spatially, the community succession was congruent across multiple sampling sites (Figure 3B), regardless of their distance from injection sites.

### 3.3. Effects of nutrition on microbial communities

#### 3.3.1. Differences in community structure at sampling sites

**Figure 4:**
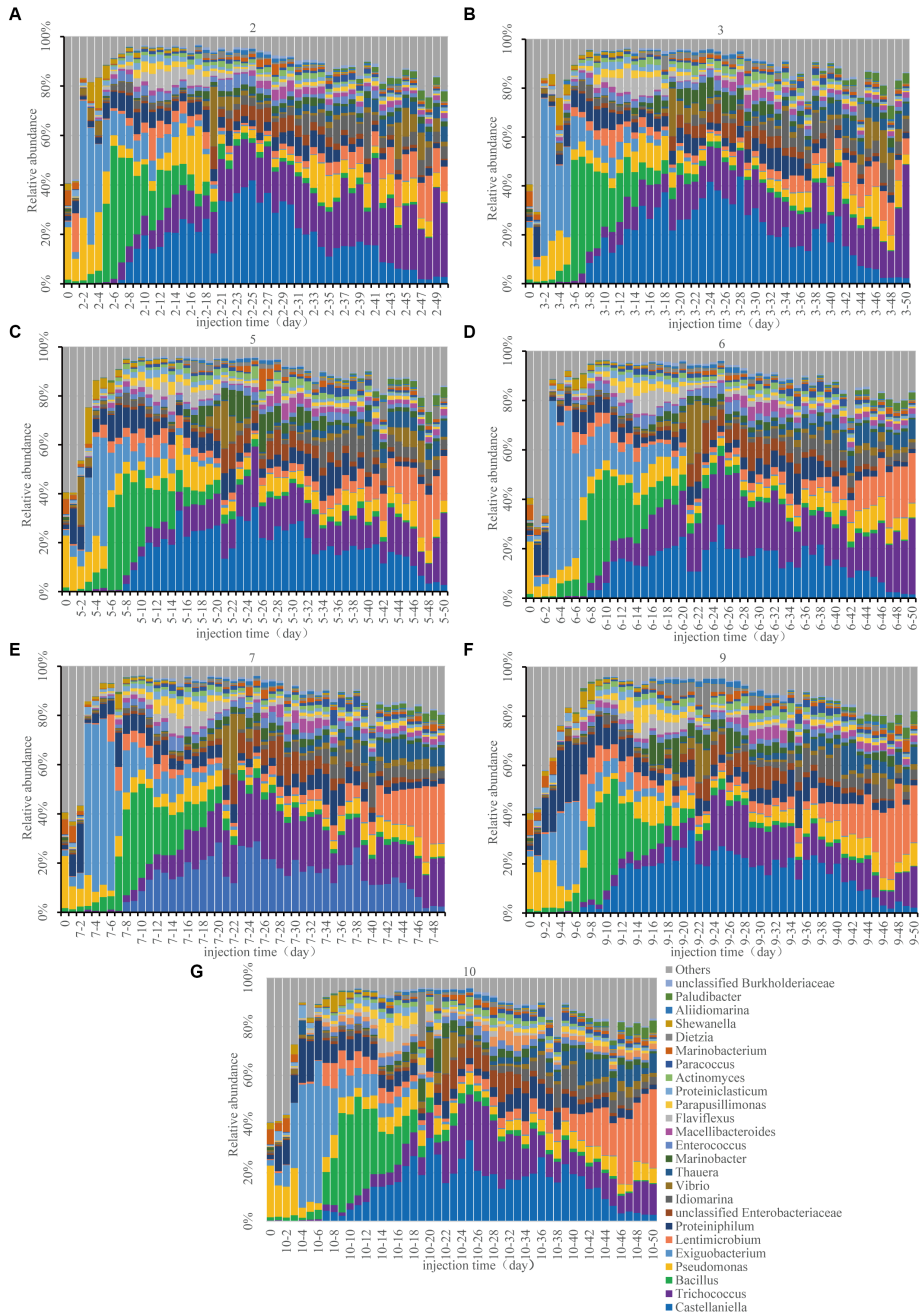
Bacterial composition changes at the genus level in the seven sampling sites. 2 **(A)**, 3 **(B)**, 5 **(C)**, 6 **(D)**, 7 **(E)**, 9 **(F)**, and 10 **(G)**. For example, 2–4 represents sampling site 2, day 4 sample.

Monitoring bacterial compositions aids in comprehending nutrient stimulatory processes. The co-occurrence network diagram of microorganisms and environmental factors is shown in Supplementary Figure S4. OTU-environmental parameters were composed of 22 nodes and 25 edges (positive edge accounted for 28%). Microbial groups exhibited varying associations with TP, TS, and TN. Firmicutes, Bacteroidetes, and Proteobacteria were the microbial groups associated with TP. The microbial groups associated with TS were composed of Firmicutes and Proteobacteria, while those related to TN contained Actinobacteria and Firmicutes. The community structure analysis of each sampling site during the monitoring period was carried out using high-throughput sequencing of the 16S rRNA gene (Figure 4). We mainly focused on the changes in dominant bacteria genera. During the monitoring period, the six predominant bacterial genera exhibiting the highest abundance include *Castellaniella*, *Trichococcus*, *Bacillus*, *Pseudomonas*, *Exiguobacterium* and *Lentimicrobium*. Most of them belonged to the Firmicutes. *Castellaniella*, which is a member of the Proteobacteria group, has the capacity to improve oil extraction by producing gas (Deng et al., 2020).

In the original mixed reservoir formation-water samples, the abundance of *Pseudomonas* from Proteobacteria was the highest, which was consistent with the monitoring result in the Xinjiang oilfield (Li et al., 2017). As a useful bacterium for petroleum production, *Pseudomonas* has the ability to synthesize surfactants, such as rhamnose-lipids, to alter the characteristics of the reservoir and crude oil (Zhao et al., 2020). On the second day following nutrient injection (approximately 0.4 PV), there was a shift in the community structure of sampling sites 2 and 3 (Figures 4A,B). *Exiguobacterium*, which belongs to the hydrocarbon oxidizing bacteria and has the capacity for alkane degradation (Deng et al., 2020), experienced significant activation. *Exiguobacterium* was the first to be activated after the injection of nutrients, which aligned with the anticipated ecological “food chain” succession in the reservoir (Ma and Chen, 2018). The second most abundant bacterium was *Pseudomonas*, which could be used as a functional bacterium for oil production (Zhao et al., 2020). The community structure of the remaining 5 sampling sites showed a similar phenomenon, with a predominance of *Proteiniphilum* and *Pseudomonas*.

On the 3rd day (about 0.6 PV), the composition of the community at sampling sites 5, 6 and 7 underwent changes (Figures 4C–E). Specifically, *Exiguobacterium* and *Pseudomonas* began dominating the community. From the 4th to 6th day (about 1 PV), *Exiguobacterium* and *Proteiniphilum* were dominant in sampling sites 9 and 10 (Figures 4F,G). *Proteiniphilum*, as an anaerobic fermentation bacterium, could promote the production of methane gas (Rs et al., 2021). *Bacillus* at sampling sites 2, 3, and 5 exhibited heavy activation. During this stage, when the injected nutrients migrated to each sampling site, hydrocarbon oxidizing bacteria (*Exiguobacterium*) were gradually activated first. Our findings indicated that differences in community structure between each sample site may be attributed to nutrient migration (Figures 2, 4).

#### 3.3.2. Community structure tends to be consistent

Microbial community structure changes were consistent across all sampling sites after approximately 20 days (about 4 PV). *Castellaniella* and *Trichococcus* were the dominant genera present at each sampling site (Figure 4). The TN content decreased significantly between day 6 and day 15, subsequently increasing (Figure 2B), which may be related to the rapid increase in oil recovery (Figure 1). At this time, the abundance of *Castellaniella* began to increase, which could produce N_2_O and N_2_ by denitrification under anaerobic or micro-oxygen conditions (Deng et al., 2020). The oil recovery potential for *Castellaniella* could be worth exploring in the future.

From the 30th day onwards, despite a reduction in injected nutrient content, the community structure of each sampling site remained almost the same (Figure 4). The abundance of *Lentimicrobium* was slightly increased. *Lentimicrobium* is a kind of strictly anaerobic microorganism, and the main fermentation products are formate and hydrogen (Sun et al., 2016), which may provide conditions for methanogens to grow. *Trichococcus* was increasing in abundance at each sampling site. As a facultative anaerobe, it has the potential to degrade alkanes (Sun et al., 2016).

In conclusion, in the long core microbial flooding simulation experiment, hydrocarbon oxidizing bacteria (*Exiguobacterium*) were first activated, and then *Bacillus* was activated. Secondly, a significant activation of *Castellaniella* capable of denitrification and gas production, and *Trichococcus*, which performed fermentation, had been observed. *Lentimicrobium* was also gaining a certain advantage. Interestingly, this was consistent with the hypothesized succession of a microbial ecological “food chain” in the reservoir, which preliminarily supported the two-step activation theory for reservoir microbes transitioning from aerobic to anaerobic states. The observed phenomenon seemed to be the norm of the microbial flooding experiment. Methanogens were not detected in our results, which may be attributed to the introduction of nitrate. The nitrate did not open a niche for lower trophic methanogens (Jami et al., 2013).

#### 3.3.3. Comparison with the oil field

The sampling sites in this study were designed to simulate production wells in oil fields. When the nutrients injected failed to reach the end of the sand-filled tube, the community structure at every sampling site exhibited a remarkable variance (Figures 3, 4). As nutrients slowly reached each sampling location, the structure of the microbial community became more uniform, which can be linked to the oil field. Tang et al. (2012) and Skidmore et al. (2005) both found significant differences in the community structure of each production well. In the oilfield field, due to cost limitations, the nutrition injected in the field was generally seriously insufficient, and the volume of injected nutrition was far less than 1 PV. In this study, about 10 PV nutrient solution was injected. Moreover, the oilfield reservoir medium was a complex multi-media with strong heterogeneity (Lei et al., 2013). The inter-well connectivity in the reservoir medium was complicated and the pore diameter of different reservoir rocks also had a filtering effect on the injected nutrients (Dong et al., 2023). Nutrients may be used up before they reach individual production wells, most of the microbial community biomass in an oilfield reservoir ecosystem can only be derived from metabolites of the upper trophic level rather than nutrients injected into the water (Gralka et al., 2020). The nutrient supply level had an impact on the microbial community configuration within each production well, as outlined in the findings by Tang et al. (2012). In other words, nutrition may be one of the main factors affecting the microbial community structure of different production wells in the same block.

### 3.4. Potential function of reservoir microorganisms

**Figure 5:**
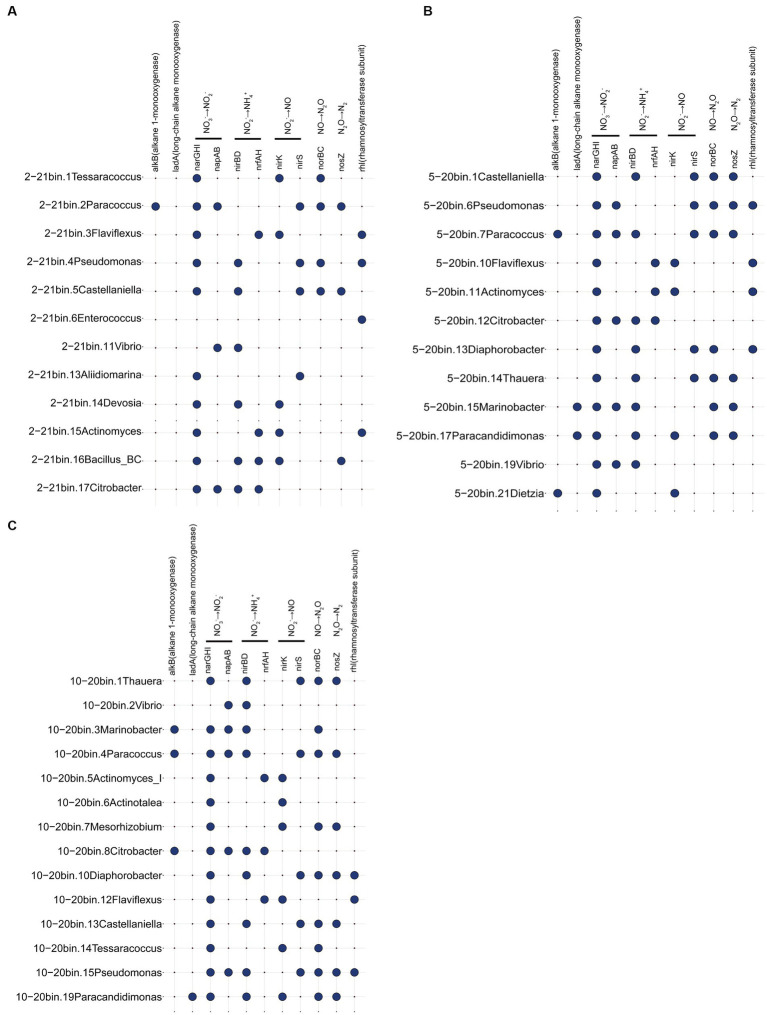
Functional genes associated with metagenomic samples 2–21 **(A)**, 5–20 **(B)**, and 10–20 **(C)**. For example, 2–21 represents sampling site 2, day 21 sample.

Metagenomic analyzes of samples from days 20 and 21 focused on genes related to the oil recovery (Figure 5). Results suggested a spectrum of reservoir microbial functionalities. The *alkB* gene was present in *Paracoccus*, *Marinobacter*, and *Citrobacter* in three metagenomic samples. Many bacteria contained the ability to produce rhamnolipids, such as *Pseudomonas*, *Flaviflexus*, and *Enterococcus*. Additionally, bacteria including *Castellaniella* and *Vibrio* were discovered that had genes involved in the nitrate reduction and denitrification processes. The results indicated that reservoir microorganisms had the potential functions of hydrocarbon degradation, denitrification gas production and surfactant production. Genes associated with SRB and methanogens (*mcrA*) were not detected in this study, which may be due to the introduction of nitrate (Jami et al., 2013). As SRB have the ability to produce H_2_S, nitrate is added to the injected water in numerous oil fields to prevent the growth of SRB and hence inhibit the formation of H_2_S (Ren et al., 2011).

Petroleum oil reservoirs are the home to phylogenetically and metabolically diverse groups of microbial communities (Liu et al., 2010). At present, this study mainly focused on the changes of reservoir microbial communities. In forthcoming research, on the one hand, the metabolites of these microorganisms need to be further explored to supplement the “food chain” of the reservoir ecosystem. On the other hand, some functional bacteria that can improve the oil recovery need to be screened. Targeted activation of these functional bacteria is helpful to enhance oil recovery.

## 4. Conclusion

This study utilized a long core simulation device to investigate the effects of nutrient injection on the microbial community in the Xinjiang oil field. Our results indicated that nutrition was one of the factors affecting the microbial communities in oil reservoirs. After nutrient injection, hydrocarbon oxidizing bacteria became active, followed by the sequential activation of facultative anaerobes and anaerobic fermenting bacteria. This was consistent with the hypothesized succession of a microbial ecological “food chain” in the reservoir, which preliminarily supported the two-step activation theory for reservoir microbes transitioning from aerobic to anaerobic states. Metagenomic results indicated that reservoir microorganisms had potential functions of hydrocarbon degradation, gas production and surfactant production. These capabilities, combined with the microbial communities’ succession, hold potential for optimized oil recovery. This work is conducive to understanding the microbial community structure in reservoirs.

## Data availability statement

The data presented in the study are deposited in the NCBI Sequence Reads Archive repository, accession number PRJNA1021155.

## Author contributions

Data processing and handling were performed by WC and HF. The manuscript was written by WC and modified by ZS, XZ, YY, XT, and DL. Project planning was conducted by TM and GL. All authors contributed to the article and approved the submitted version.

## Footnotes

^1^https://docs.qiime2.org/2019.7/tutorials/

^2^https://github.com/qiime2/q2-diversity
