# Supplementary figures and images for: Effects of nutrient injection on the Xinjiang oil field microbial community studied in a long core flooding simulation device
Source: Front Microbiol. 2023 Oct 12;14:1230274. doi: 10.3389/fmicb.2023.1230274 (PMC10602641; doi:10.3389/fmicb.2023.1230274)

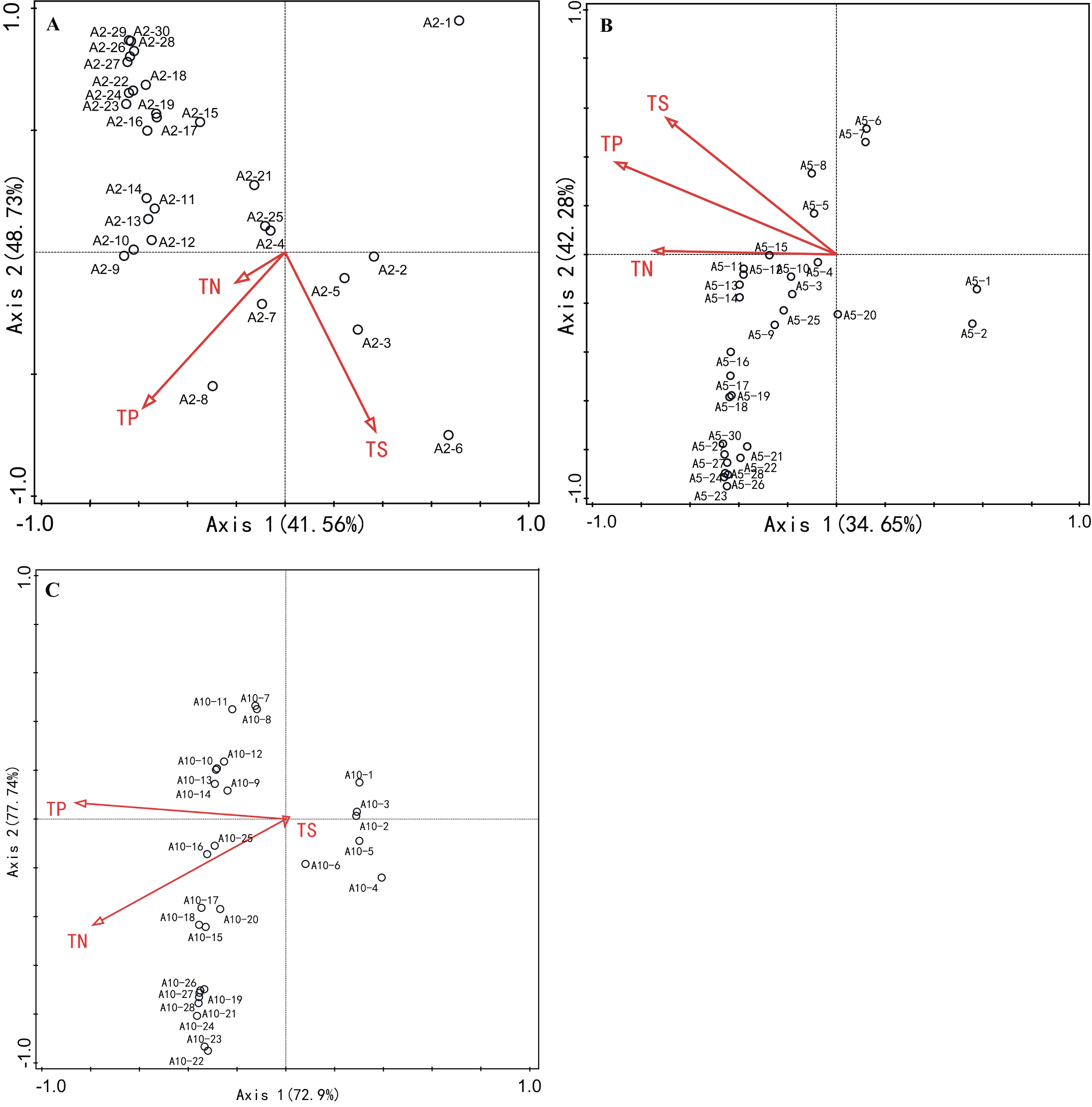

Supplement: Supplementary file 1 [file Data_Sheet_1.ZIP › Raw Data/Figure/Fig.4.tif]

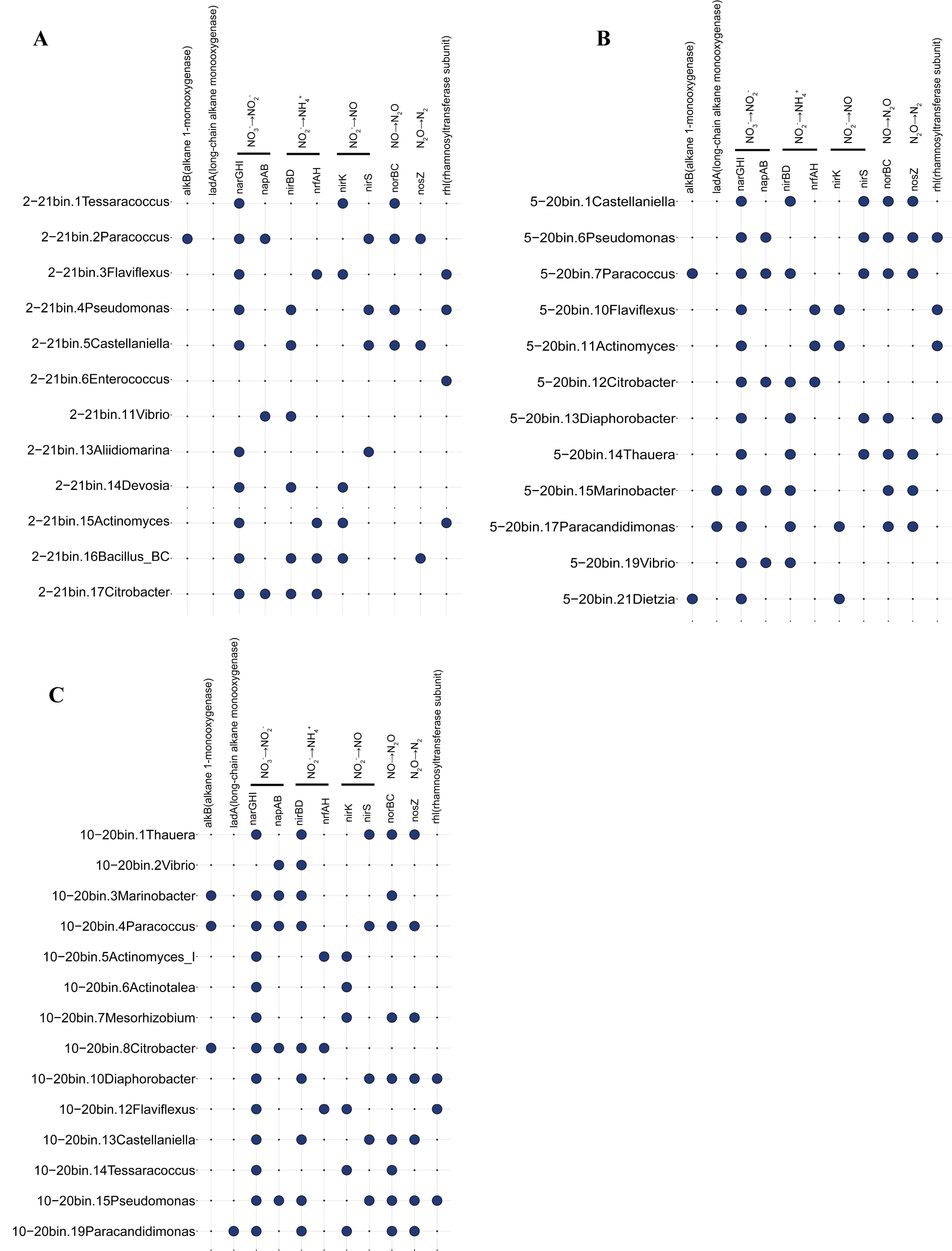

Supplement: Supplementary file 1 [file Data_Sheet_1.ZIP › Raw Data/Figure/Fig.9.tif]

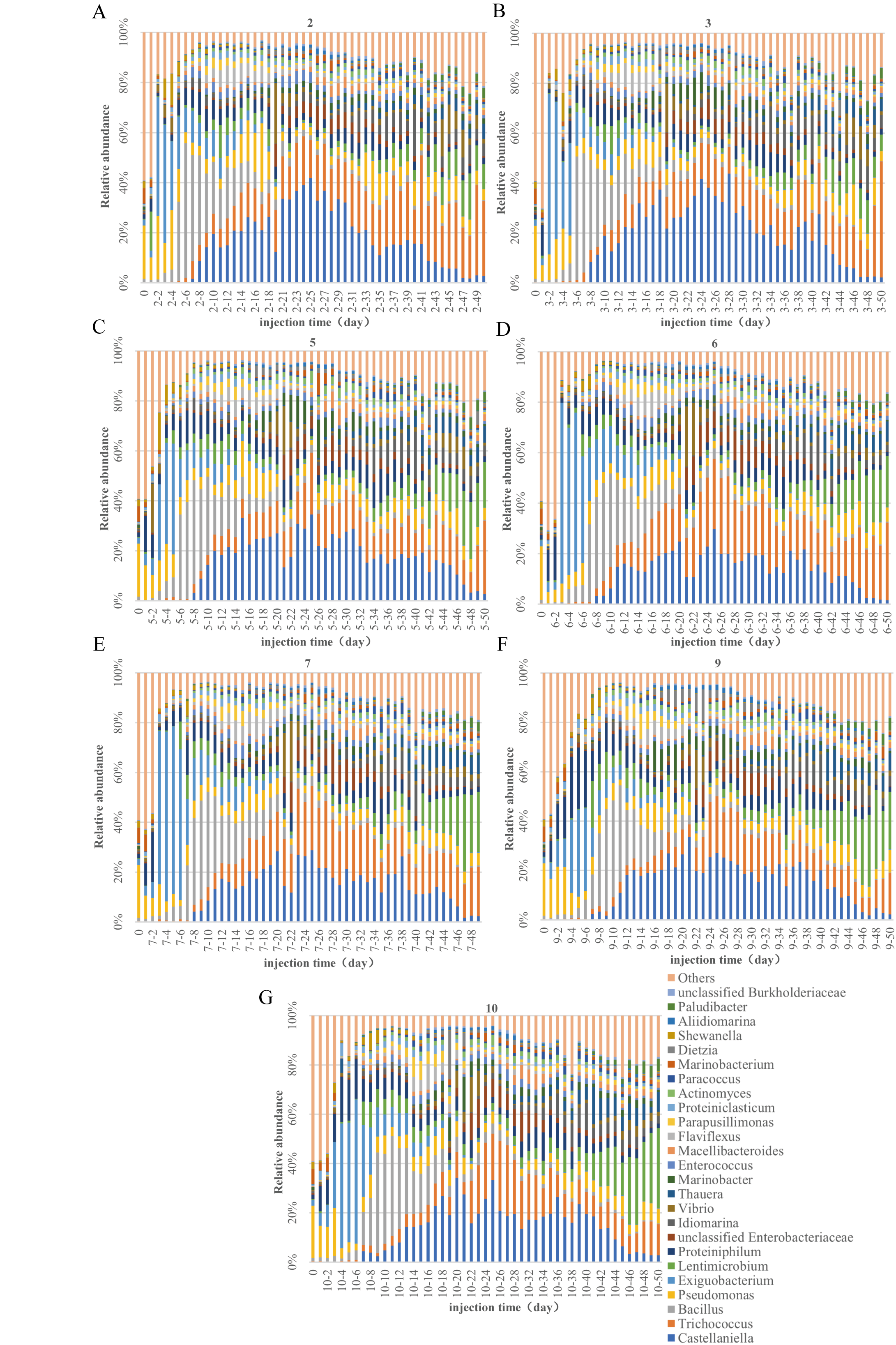

Supplement: Supplementary file 1 [file Data_Sheet_1.ZIP › Raw Data/Figure/Fig.8.tif]

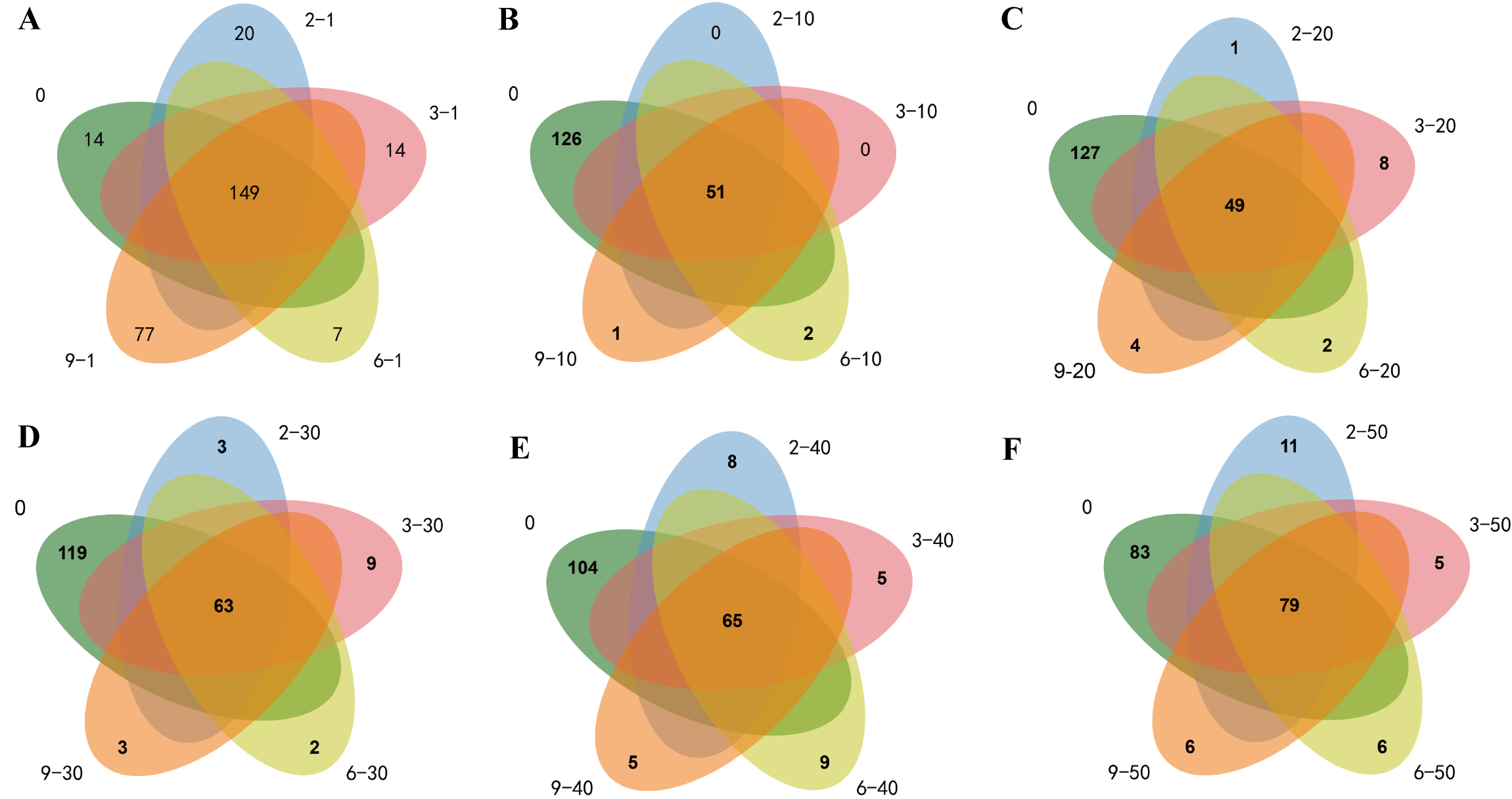

Supplement: Supplementary file 1 [file Data_Sheet_1.ZIP › Raw Data/Figure/Fig.7.tif]

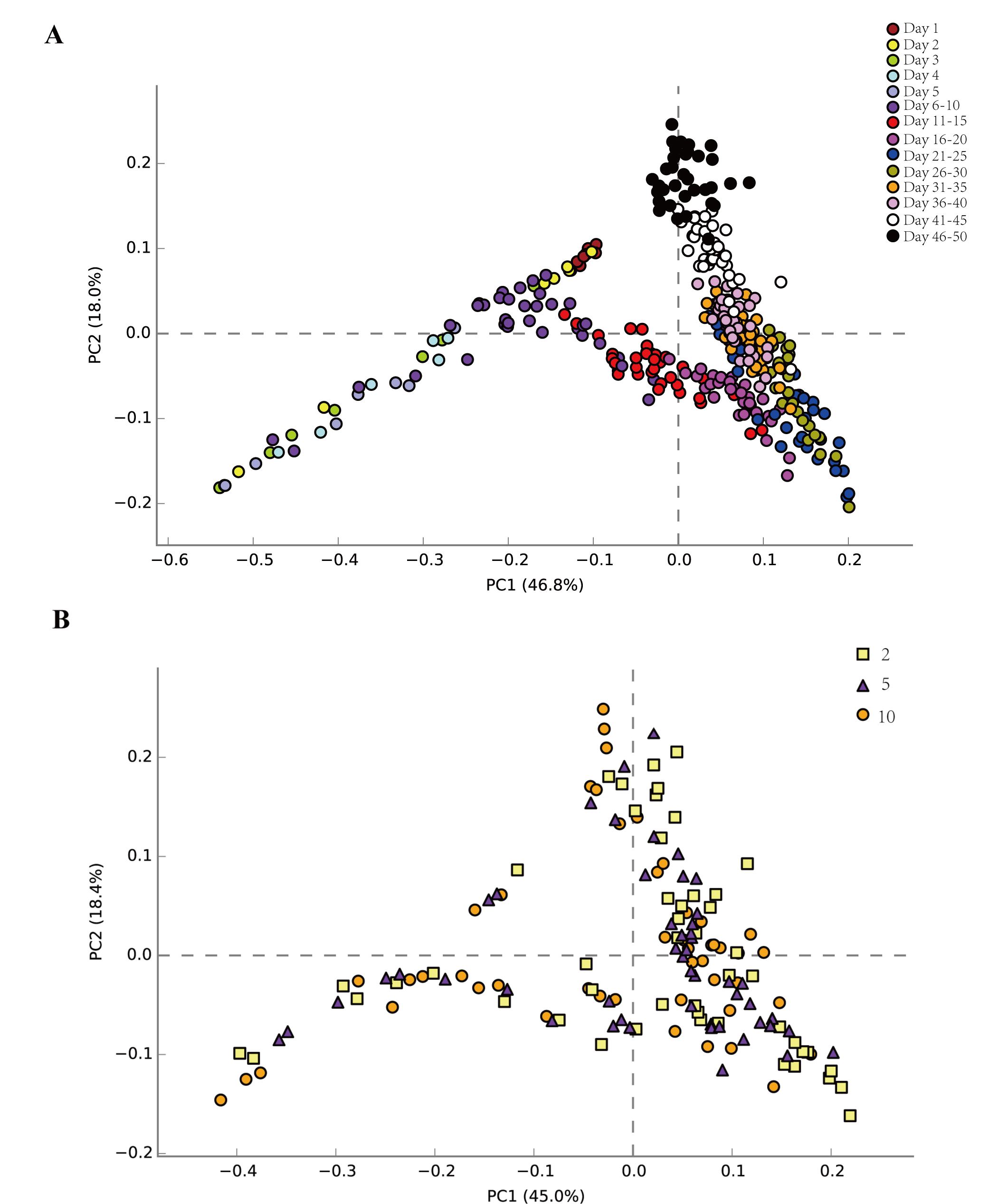

Supplement: Supplementary file 1 [file Data_Sheet_1.ZIP › Raw Data/Figure/Fig.6.tif]

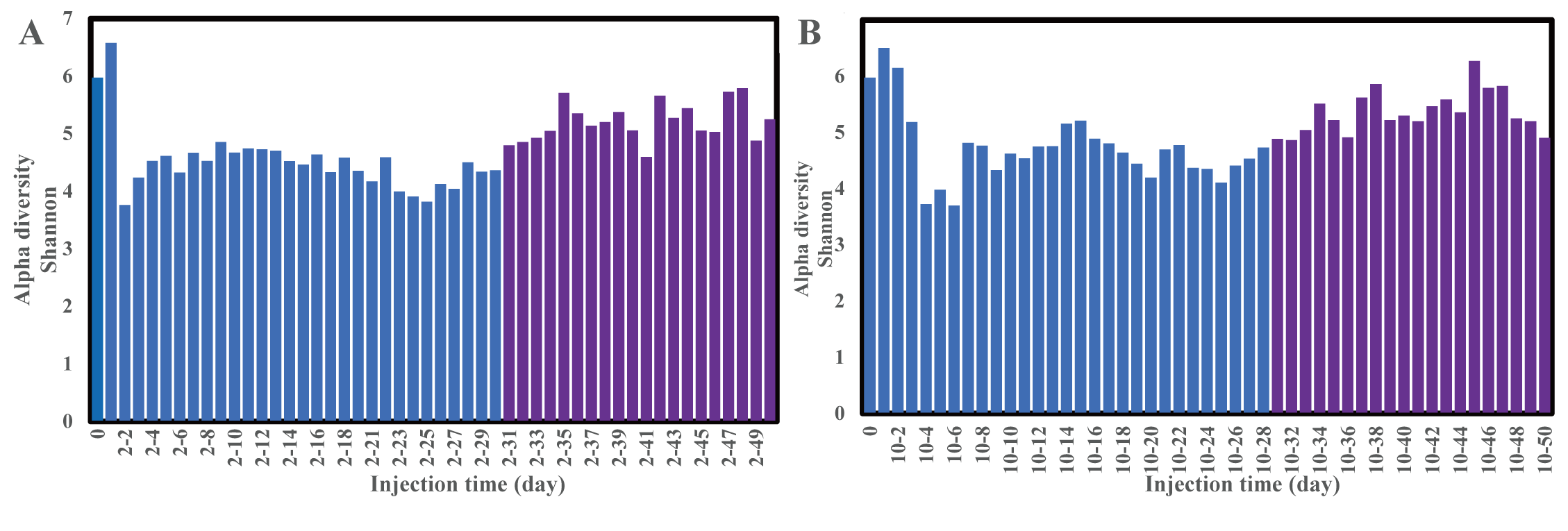

Supplement: Supplementary file 1 [file Data_Sheet_1.ZIP › Raw Data/Figure/Fig.5.tif]

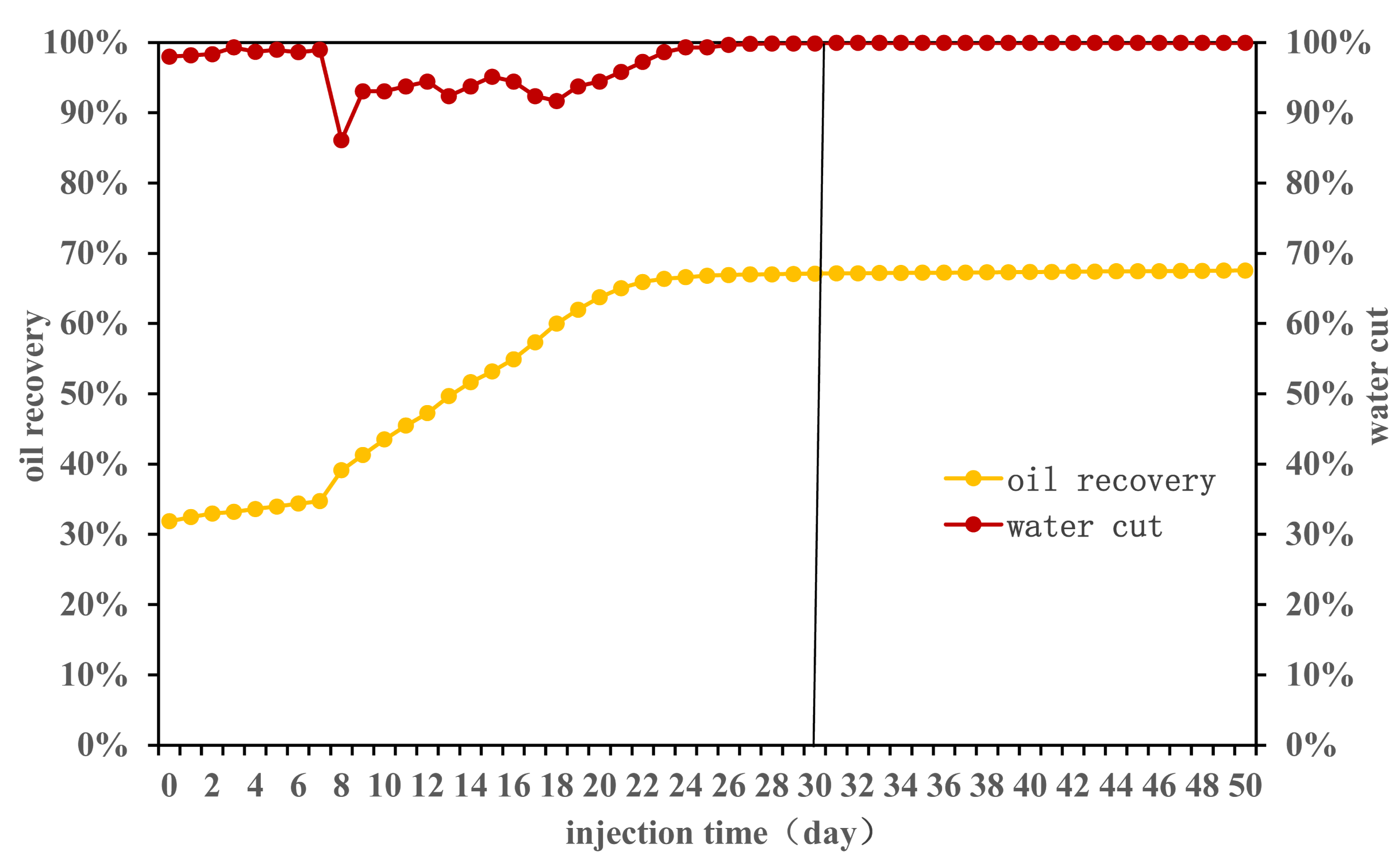

Supplement: Supplementary file 1 [file Data_Sheet_1.ZIP › Raw Data/Figure/Fig 2.tif]

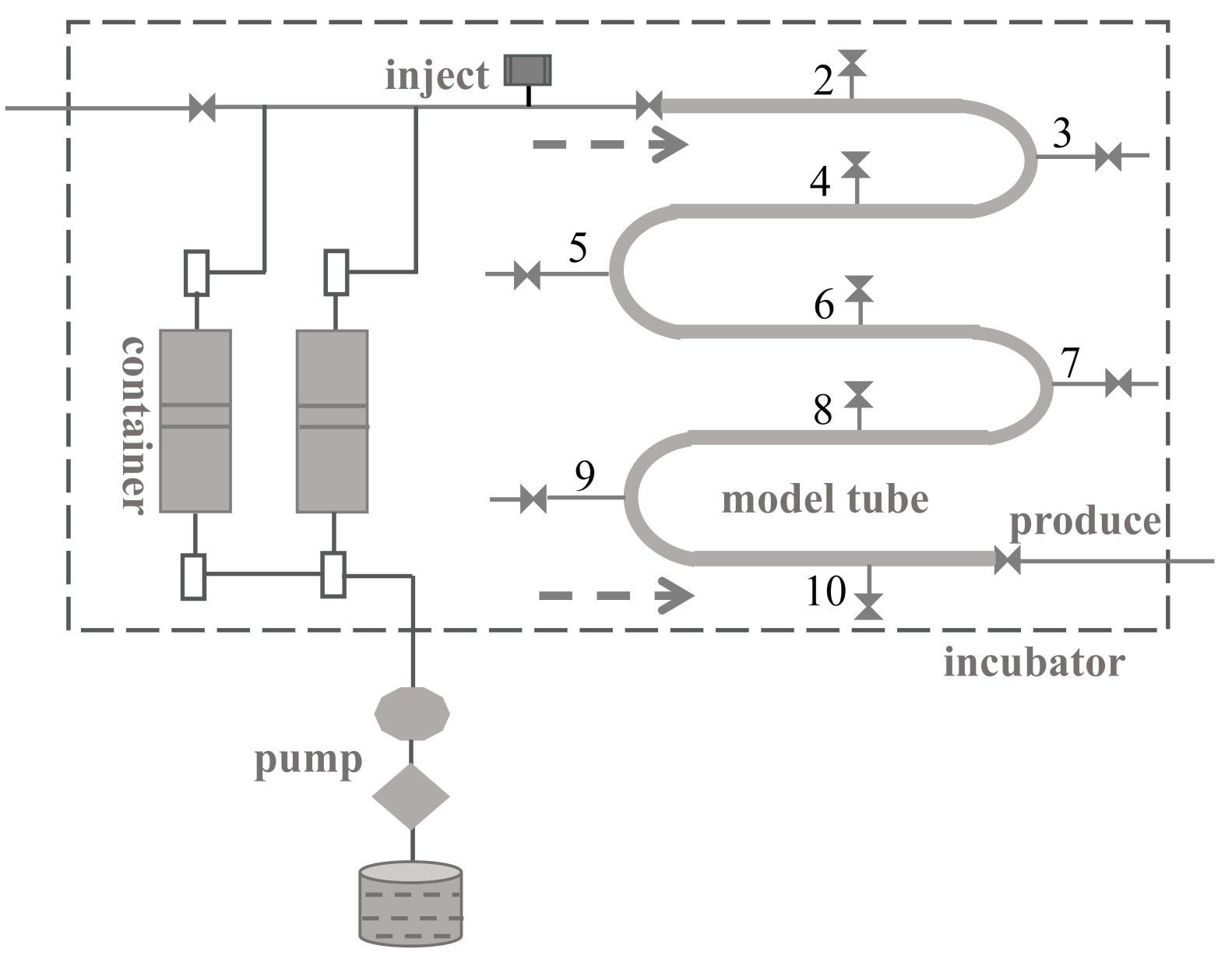

Supplement: Supplementary file 1 [file Data_Sheet_1.ZIP › Raw Data/Figure/Fig. 1.tif]

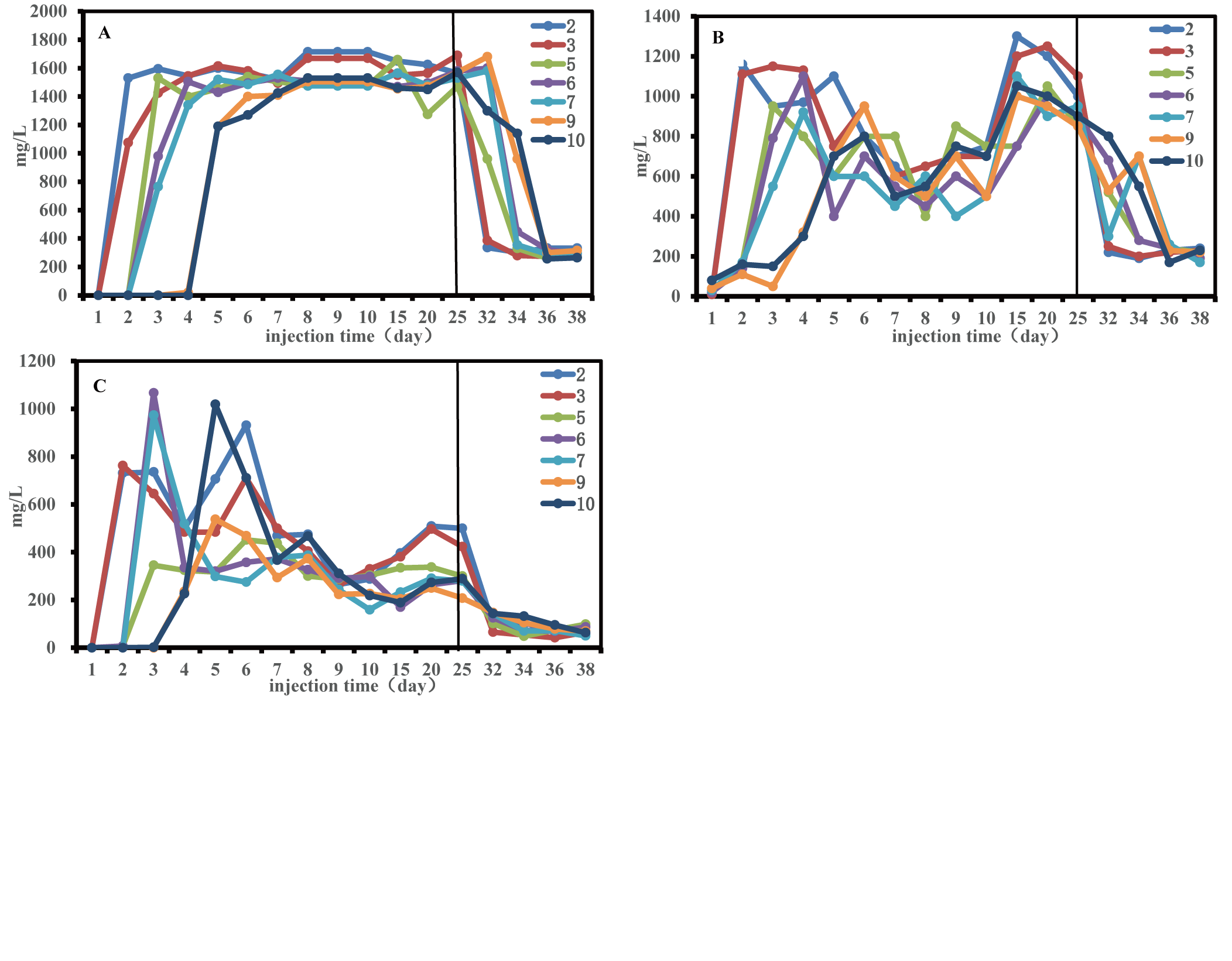

Supplement: Supplementary file 1 [file Data_Sheet_1.ZIP › Raw Data/Figure/Fig.3.tif]

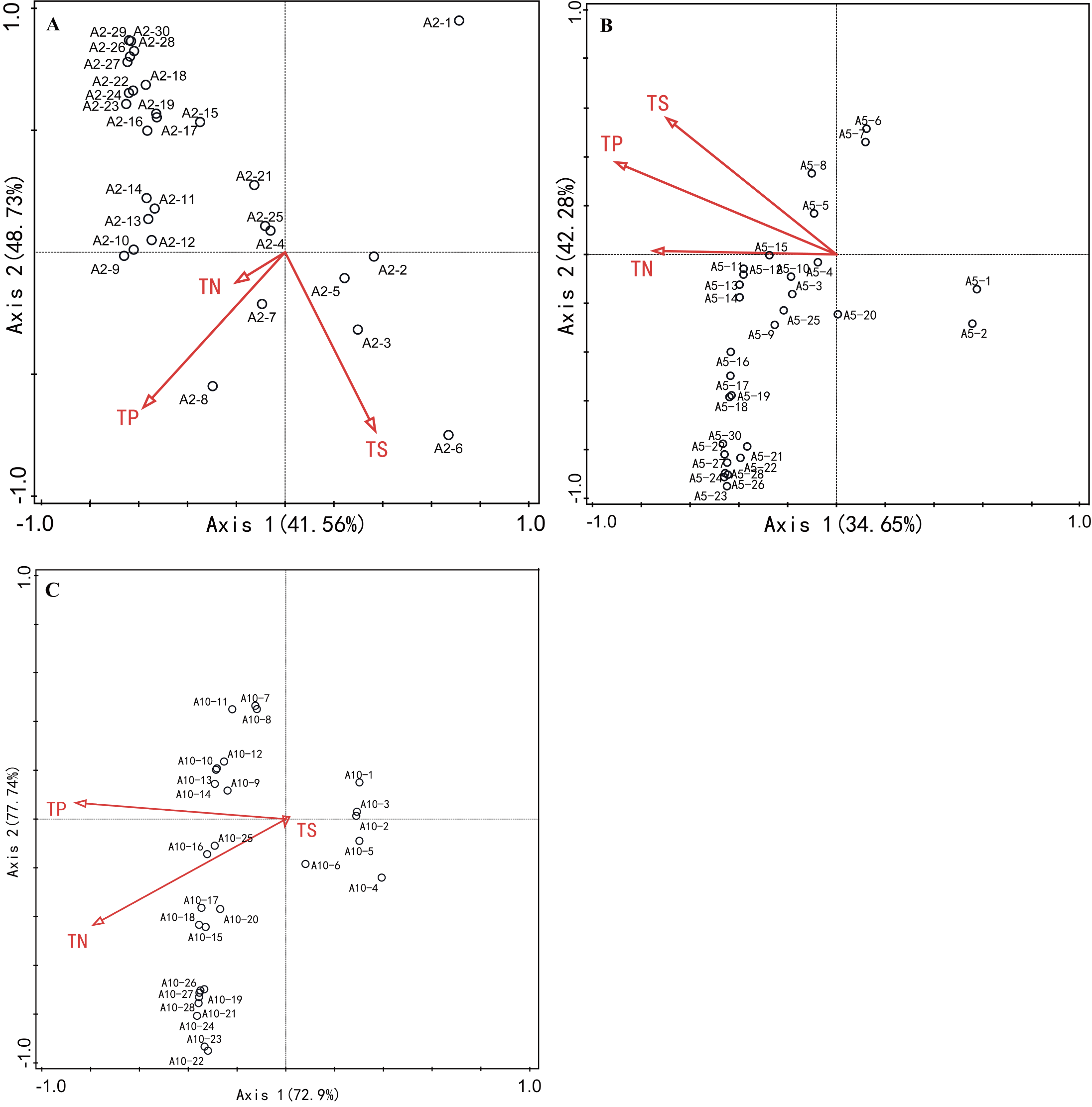

Supplement: Supplementary file 3 [file Image_1.TIF]

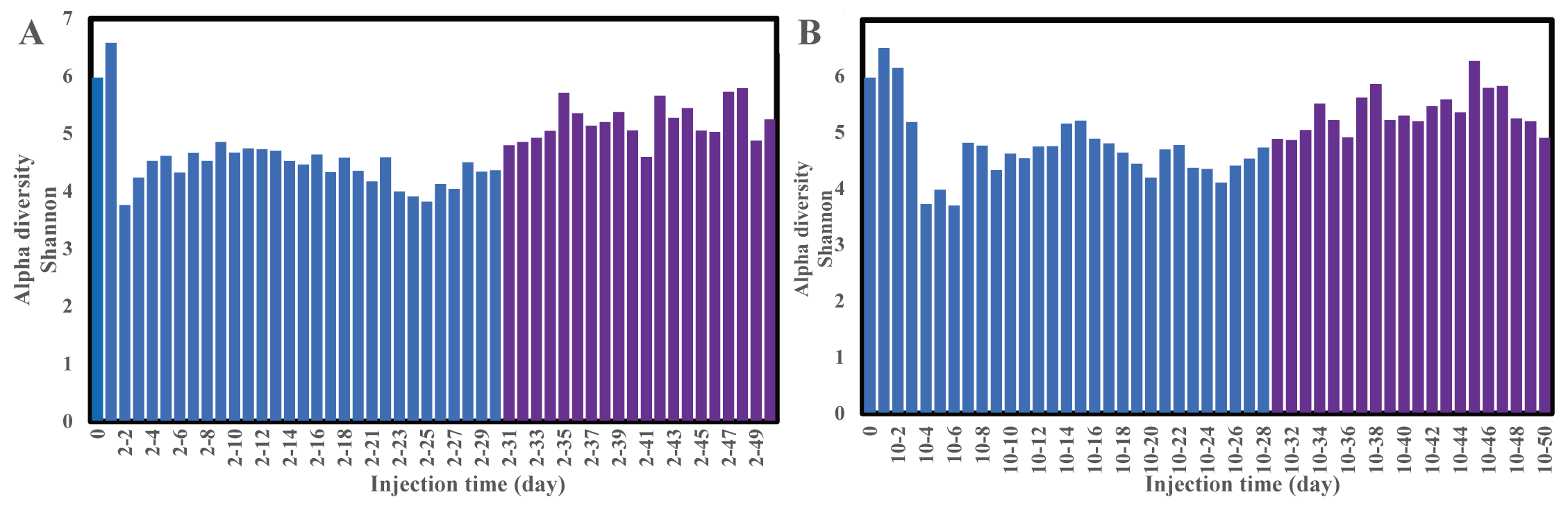

Supplement: Supplementary file 4 [file Image_2.TIF]

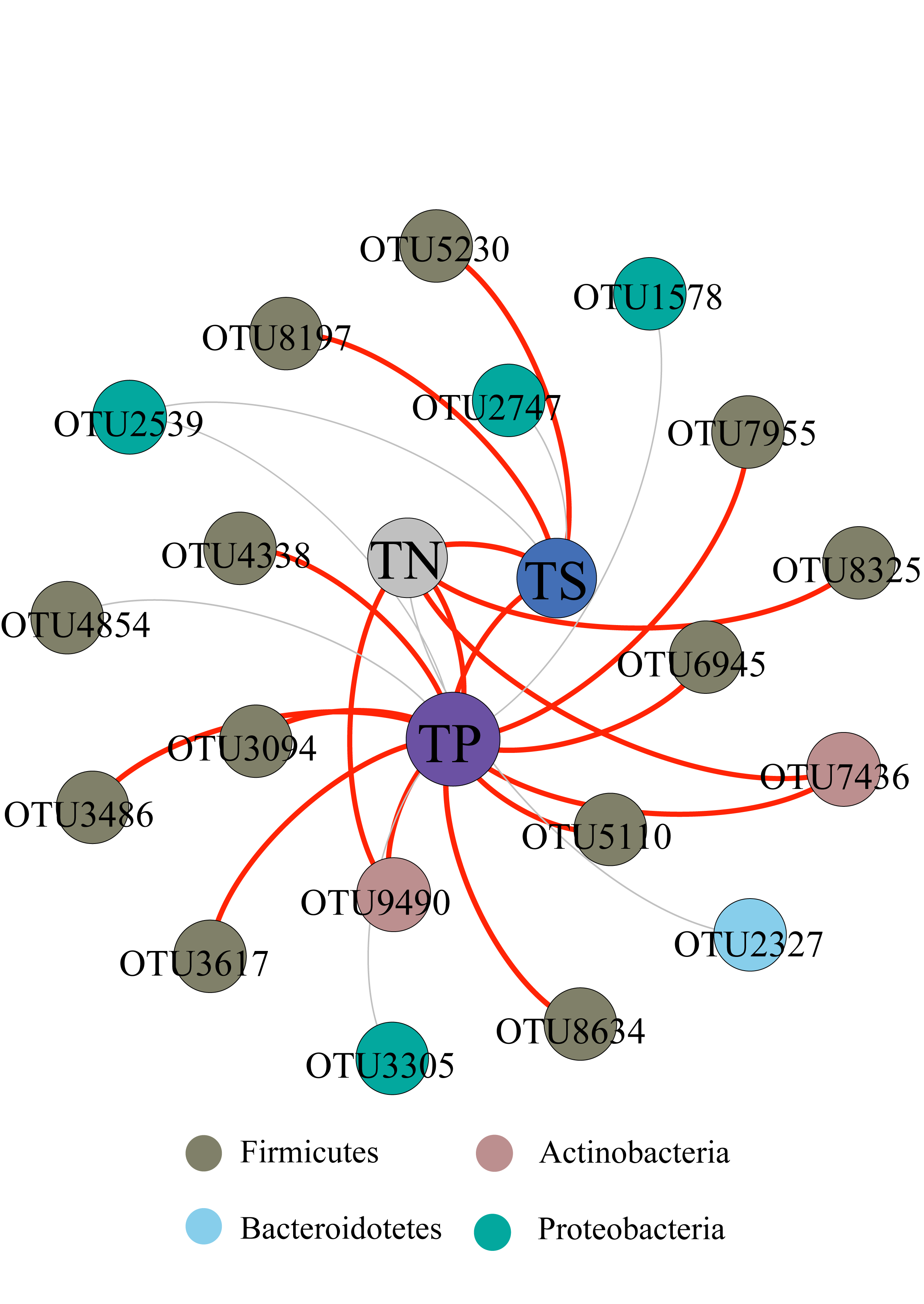

Supplement: Supplementary file 5 [file Image_3.TIF]

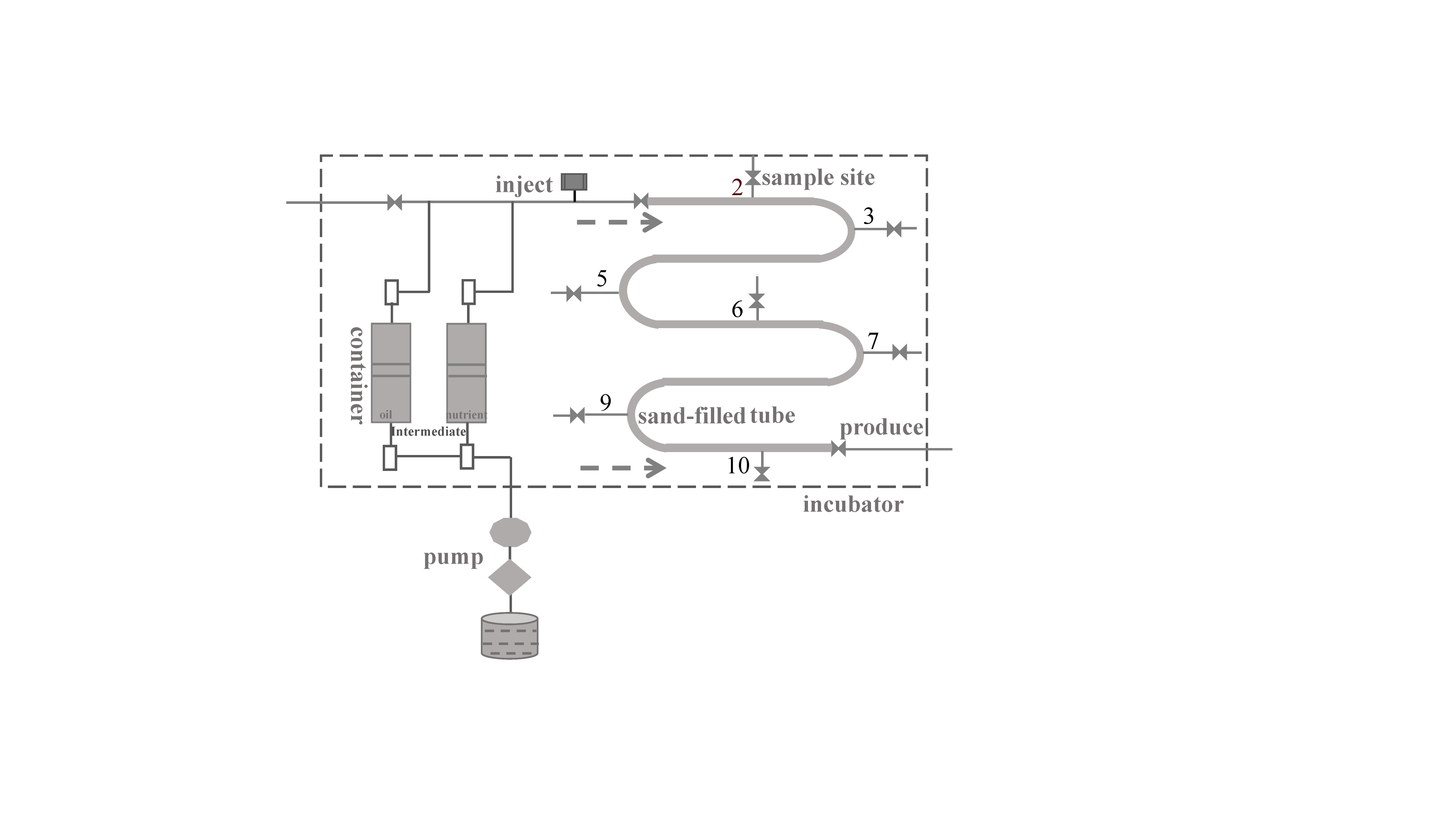

Supplement: Supplementary file 6 [file Image_4.TIF]
