# Supplementary material for: Effects of nutrient injection on the Xinjiang oil field microbial community studied in a long core flooding simulation device
Source: Front Microbiol. 2023 Oct 12;14:1230274. doi: 10.3389/fmicb.2023.1230274 (PMC10602641; doi:10.3389/fmicb.2023.1230274)
Supplement: Supplementary file 2 [file Data_Sheet_2.PDF]

Effects of nutrient injection on the Xinjiang oil field microbial community studied in a long core flooding simulation device

Wei Cheng[1], Huiqiang Fan[1], Yuan Yun[1], Xueqing Zhao[1], Zhaoying Su[1], Xuefeng Tian[1], Dakun Liu[1], Ting Ma[1,2*], Guoqiang Li [1,2*]

*For correspondence author

E-mail: tingma@nankai.edu.cn, Tel. 86-22-23508870; E-mail: gqli@nankai.edu.cn,

Tel. 022-23498185.

1 Supplementary Figures and Tables

1.1 Supplementary Figures

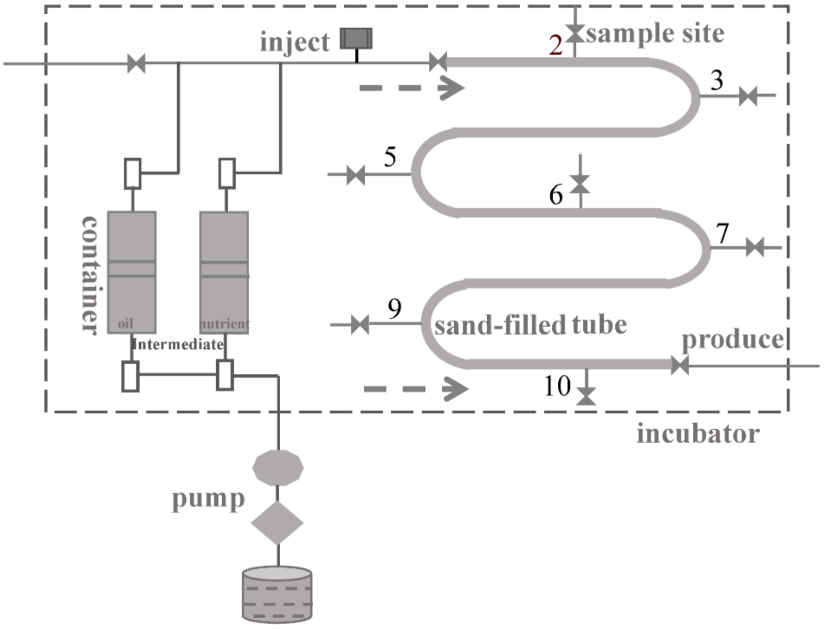

Fig. S1 A scheme of the long core microbial flooding simulation device.

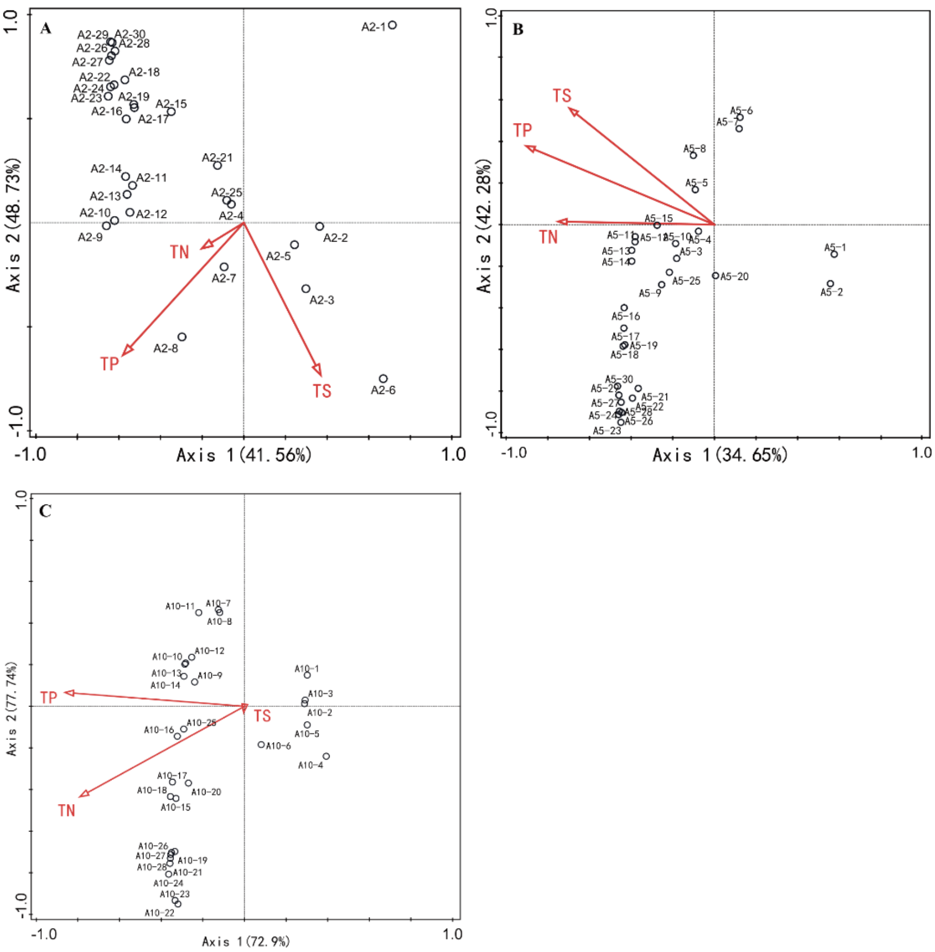

Fig. S2 Correlation analysis of bacterial community composition and environmental factors in the extracted liquid from sampling site 2 (A), 5(B), 10(C) in the first 30 days. Total phosphorus (TP); Total sugar (TS); Total nitrogen (TN).

Table S3 Effects of total sugar (TS), total nitrogen (TN) and total phosphorus (TP) on bacterial community composition in three sampling sites (2, 5 and 10). The table shows the p-values for each selected environmental factor.

Sampling site	Environmental factor	P value
2	TS	0.272
	TN	0.550
	TP	0.004
5	TS	0.096
	TN	0.109
	TP	0.016
10	TS	0.650
	TN	0.004
	TP	0.002

$P < 0.05$: environmental factors have significant effects on community structure.

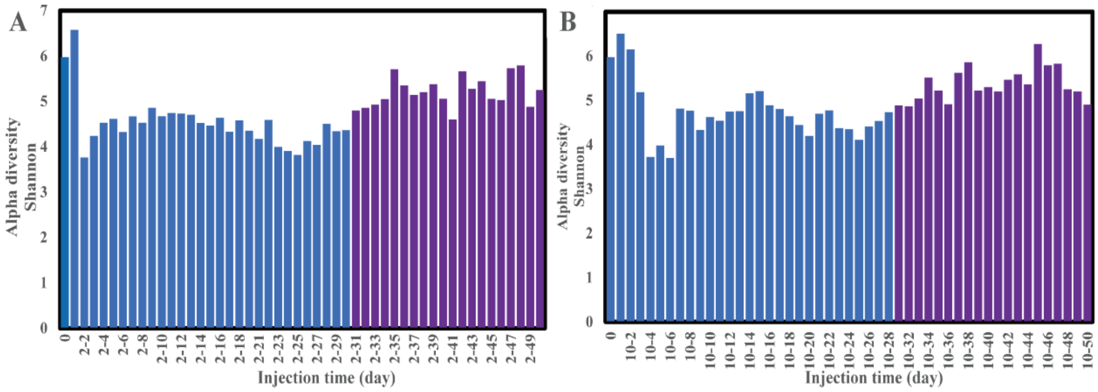

Fig. S3 Changes in alpha diversity of bacterial community in produced fluid at sampling sites 2 (A) and 10 (B). For example, 2-4 represents 2 sampling site, day 4 sample alpha diversity.

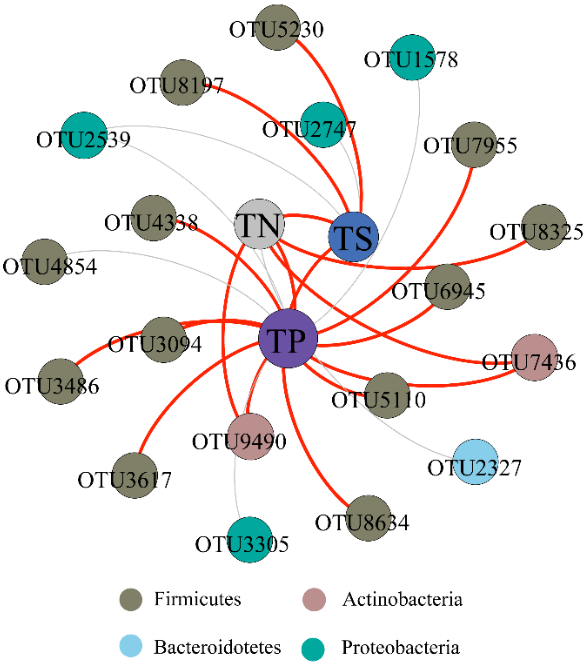

Fig. S4 The network visualizes the interactions between environmental factors and microbes during the experiment. Negative correlations are shown in red and positive correlations are shown in gray. Total phosphorus (TP); Total sugar (TS); Total nitrogen (TN). Interaction between OTU and environmental factors (Spearman's |r| N> 0.7, p-value<0.05).

1.2 Supplementary Tables

Table S1 Composition of inorganic salt ions in mixed well water samples（$mg \cdot L^{-1}$）

Ion species	PO_4^{3-}	SO_4^{2-}	NO_3^-	Ca^{2-}	Mg^{2+}	Na^+	K^+
content	14.63	8.07	10.15	100	52.4	3280	284

Table S2 Standard curve making procedures

Tube number	0	1	2	3	4	5	6	7	8
H_2O（mL）	2.0	1.6	1.4	1.2	1.0	0.8	0.6	0.4	0.2
Glucose solution（mL）	0.0	0.4	0.6	0.8	1.0	1.2	1.4	1.6	1.8
6% Phenol（mL）	1.0	1.0	1.0	1.0	1.0	1.0	1.0	1.0	1.0
H_2SO_4（mL）	5.0	5.0	5.0	5.0	5.0	5.0	5.0	5.0	5.0

We use phenol-sulfuric acid method to determine the sugar in the sample.

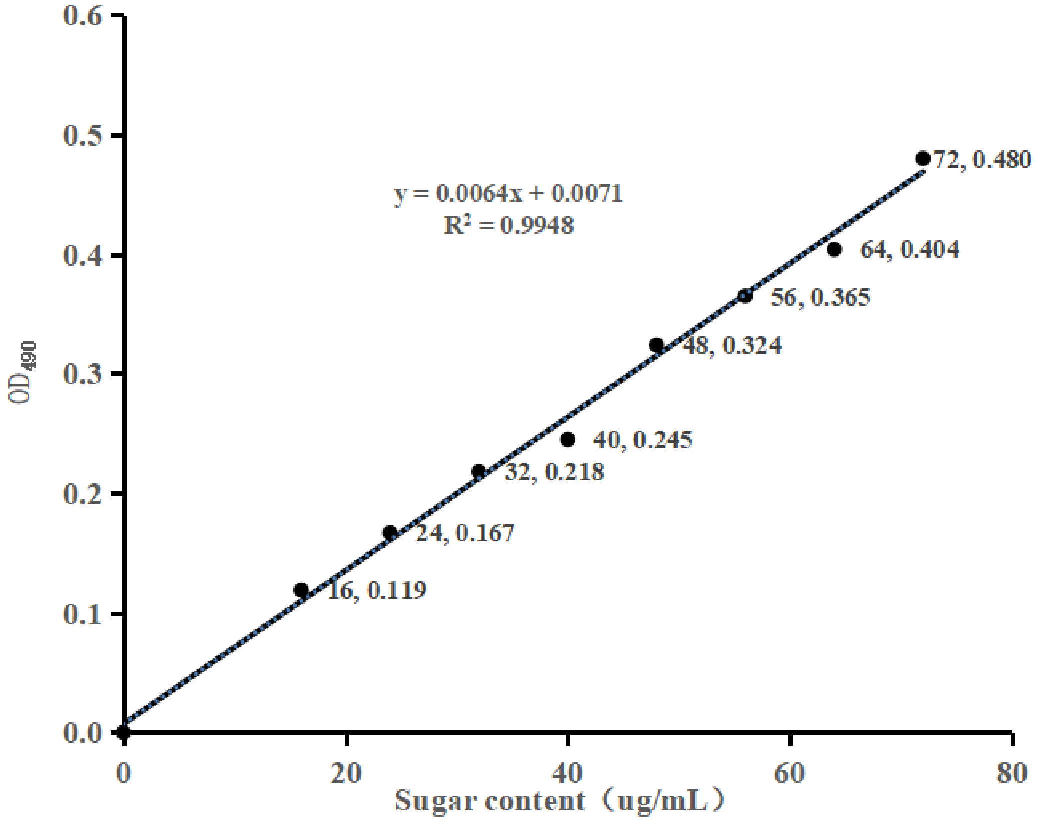

Fig. 2 Standard curve for the determination of total sugar.

2 Supplementary methods

2.1 The total nitrogen content in the samples was determined by persulfate oxidation.

(1) Open the DBR digester and heat it to 105 °C.
(2) Add total nitrogen persulfate reagent into the low range digestion reagent tube.
(3) Sample determination: Add 2 mL sample into a reagent tube (estimate the sample concentration and dilute it with deionized water in the kit to the measurement range). A 2 mL kit of deionized water was used as a blank control.
(4) Cover and shake vigorously for 30 seconds.
(5) Insert the test tube into the digestion device and heat digestion for 30 minutes.
(6) Remove and cool to room temperature after digestion.
(7) Add total nitrogen A reagent.
(8) Cover and shake for 15 seconds and let stand for 3 minutes.
(9) At the end of the reaction, total nitrogen B reagent was added.
(10) Cover, shake the reagent tube up and down violently for 15 seconds, and let it stand for 2 minutes.
(11) After the reaction, open the total nitrogen C reagent tube and add 2 mL digestion solution.
(12) Cover and shake for 10 seconds. Let stand for 5 minutes.
(13) The time is over and the reading is measured.

2.2 Determination of total phosphorus in samples by digestion-Molybdenum-antimony method.

(1) Open the DBR digester and heat it to 150 °C.
(2) Add 5 mL sample into the total phosphorus reagent tube with a pipette (estimate the sample concentration and dilute it to the measuring range with deionized water in the kit). A 5 mL kit of deionized water was used as a blank control.
(3) Potassium Persulfate reagent was added into reagent tube.
(4) Cover the lid and shake vigorously to dissolve the powder.
(5) Insert the test tube into the digestion device and heat digestion for 30 minutes.
(6) Immediately after digestion, take out the reagent tube and cool it to room temperature.
(7) Open the reagent tube and add 2 mL of 1.54N sodium hydroxide solution.
(8) Add 0.5 mL molybdovanadium heteropolysate reagent into the reagent tube and react for 7 minutes.
(9) After the time is over, clean the test tube and measure the reading.

2.3 DNA extraction methods

(1) Weigh 0.5g glass beads in the grinding tube, and add Buffer SLX MLUS 800 to the evenly mixed sample in advance, and shock it (speed 6.0, cycle 5, time 1min, interval 1min30s).
(2) Add 80 microliter Buffer DS, vortex uniform (10 s).
(3) 70°C water bath temperature for 10 minutes, including vortices every 5 minutes.
(4) Centrifuge 12000 g for 5 min. Transfer all the supernatant into the new 2 mL EP

tube, and follow the kit instructions for remaining steps after sample pretreatment.

Gas permeability calculation formula

$$\text{Permeability}=2*E/60*0.1*\$H\$4*C*0.1/((3.14*B^{2}/4)*((D+0.1)^{2}-0.01))$$

E: Flow rate of nitrogen through long sand tube Qg (mL/min)
H: Nitrogen viscosity CP(mpa.s)
C: Long sand pipe length L(cm)
B: Long sand pipe diameter D(cm)
D: Nitrogen pressure at the injection end of the long sand filling pipe (Pg/MPa)
